# 

**Published:** 1893-08

**Authors:** 


ESTABLISHED 1855. SUBSCRIPTION, $2.00.

LIBRARY
Indexed
S. G. O.

### ATLANTA MEDICAL AND SURGICAL JOURNAL.

OLD SERIES, VOL. XXVI.
NEW SERIES, VOL. X.
ATLANTA, GA., AUGUST, 1893. No. 6.

LUTHER B. GRANDY, M. D.,

MANAGING EDITOR.

M. B. HUTCHINS, M. D.,

BUSINESS MANAGER.

